# Phytoremediation, Bioaugmentation, and the Plant Microbiome

**DOI:** 10.1021/acs.est.2c05970

**Published:** 2022-11-18

**Authors:** Reid A. Simmer, Jerald L. Schnoor

**Affiliations:** Department of Civil and Environmental Engineering, IIHR Hydroscience & Engineering, The University of Iowa, Iowa City, Iowa 52242, United States

**Keywords:** Phytoremediation, bioremediation, bioaugmentation, microbiome, rhizosphere, groundwater

## Abstract

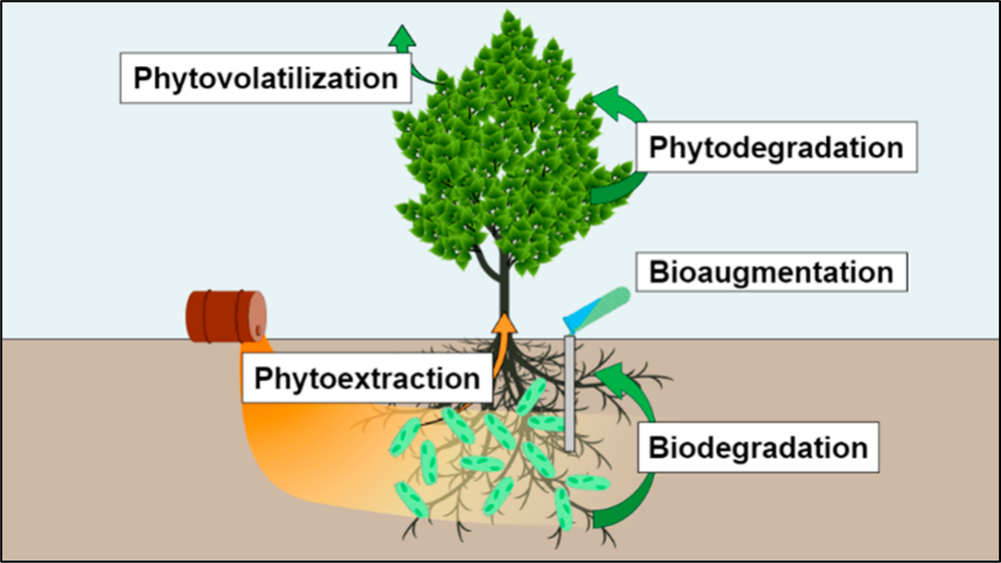

Understanding plant biology and related microbial ecology as a means to phytoremediate soil and groundwater contamination has broadened and advanced the field of environmental engineering and science over the past 30 years. Using plants to transform and degrade xenobiotic organic pollutants delivers new methods for environmental restoration. Manipulations of the plant microbiome through bioaugmentation, endophytes, adding various growth factors, genetic modification, and/or selecting the microbial community via insertion of probiotics or phages for gene transfer are future areas of research to further expand this green, cost-effective, aesthetically pleasing technology—phytoremediation.

## Abstract 2

A perspective on phytoremediation, its promise, and how it developed into a powerful green technology for cleaning hazardous waste sites.

## Introduction

Phytoremediation, the modern use of plants to help clean the environment, began in the early 1980s as a research area with studies on the uptake of metals by hyperaccumulating plants^1^ and the toxicity of pesticides to crop and nontarget plants.^2^ However, in fact, the history of plants to improve soil quality goes back millennia to Greek and Roman times when fava beans (legumes) were used to provide soil cover and nitrogen fertilization for vineyards. In northern Europe in the 1700s, lupine was planted to improve poor quality sandy soils by adding organic carbon and nutrients via nitrogen fixation and root turnover.^3^ Likely, these were the first plants grown for land remediation (phytoremediation) purposes. However, plants were unknown for their potential to remediate contaminated soil and hazardous waste sites until recently.

Considering that most of the land on Earth is covered by plants, it stands to reason that they influence the fate and transport of chemicals and xenobiotic compounds to a large extent. Twenty-nine percent of the Earth’s surface is land, and most of that land, 71%, is habitable (the remainder is glaciated or barren).^4^ Of habitable land, plants cover 98%, consisting of 50% in agriculture, 37% in forests, and 11% in shrubs and grassland.^4^ Trees and forests account for roughly one-half of all primary production and carbon sequestration. Moreover, because of their enormous biomass, plants represent the greatest oxidative enzymatic power on Earth, with catabolic enzyme systems evolved for respiration, detoxification, and plant protection that can fortuitously biodegrade many toxic organic chemicals.

Remarkably, plants comprise 82% of all living biomass on Earth, 450 billion metric tons of carbon (out of 550 GtC).^5^ They capture carbon dioxide from the atmosphere to partially offset human greenhouse gas emissions, and they photosynthesize 100 billion metric tons of carbon per year (100 GtC/year), which serves as food for all living things. It is no wonder that bacteria, fungi, and an entire ecosystem of decomposers reside in, on, and around plants as nature’s primary producers of carbon substrate (food). The next largest category of biomass on Earth is bacteria (∼10% of all living biomass), while human biomass is relegated to a paltry 0.01%.^5^

## Phytoremediation Background

**Figure 1:**
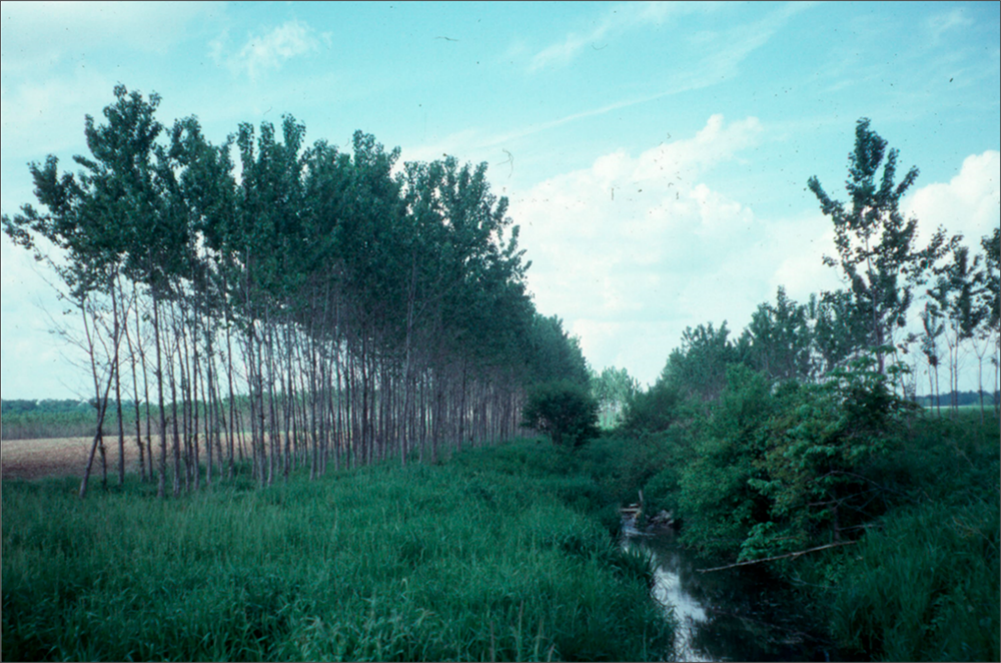
Hybrid poplar plantation and riparian zone buffer strip from the Ph.D. research of Louis Licht at Amana, Iowa. Photo was taken in 1997, approximately 7 years after planting.^6−8^

As authors, we credit many researchers, students, postdoctoral fellows, and consulting firms since the 1980s with unraveling the mysteries of phytoremediation and employing it at thousands of hazardous waste sites. At the University of Iowa, an energetic and creative Ph.D. student, Louis Licht, was the first to see the potential of “phyto”.^6^ We called it “vegetative remediation” at the time, for lack of a better moniker.^7,8^ We worked especially with hybrid poplar (*Populus* spp.) as buffer zones along stream margins to intercept nutrients and biodegrade pesticides before they impacted water quality (Figure 1).

Why poplar? Although we employed many different plants, including trees, grasses, and wetland species, most of our research dealt with hybrid poplar trees as model plants because of their incredible versatility and deep-rooting capability. Poplar trees (*Populus* spp.) are extremely fast growing (5–8 ft per year, 7.5 tons of dry matter per acre per year) and can flourish geographically from boreal midcontinental regions to subtropical zones. They transpire large quantities of water (up to 100 gal per mature tree each day) when it is available, thus exerting some hydraulic control on soluble soil and groundwater contaminants. Poplars can withstand flooded conditions for short periods (a couple of weeks to months), but generally, their roots need to remain aerobic. This is facilitated by aerenchyma, gas passages through the vascular structure, which allows poplar to transport oxygen downward to maintain root systems and the rhizosphere. Thousands of hybrid poplar cultivars are commercially available throughout the world, mostly developed by traditional plant breeding techniques. They can be clonally propagated. We plant genetically identical male clones because most property owners do not want the airborne “cotton”, which disperses the seeds from female poplar trees. Poplars are among tree species that can undergo coppicing, that is, they can grow back from a cut stump more vigorously, leaving the perennial roots in place and producing a bushier, more productive plant. Lastly, *Populus* is considered a “model plant” because the entire genome has been sequenced and is mostly annotated.^9^ It is also a model plant in the sense that poplars are widely utilized by phytoengineers at actual contaminated sites as the species of choice to transpire water and to facilitate biodegradation of contaminants. Often, nothing will grow at these sites, yet the hardy poplar can be viewed as an instrument of phytoremediation and also as the first step in improving poor soils (adding carbon and nitrogen from root turnover) such that native species can eventually be planted to restore the ecology of the site.

Pesticides in runoff were the first obvious target compounds for research because agrochemical companies had not published the uptake and active mechanisms of herbicides and insecticides for proprietary reasons.^7,10^ In 1982, Briggs, Bromilow, and Evans studied the physical chemistry for uptake of nonionic organic chemicals by barley.^2^ However, it was not until 1994 that researchers realized that plant uptake and metabolism of xenobiotic chemicals was analogous to metabolism in higher organisms. Sandermann proposed the “green liver” model for plant metabolism of organics.^11^ Coleman developed the concept further emphasizing three phases of biotransformation and detoxification. Phase I: activation to a more polar metabolite such as hydroxylation by cytochrome P450 monooxygenases. Phase II: conjugation by enzymes such as glutathione-*S*-transferase (GST), glucuronosyltransferases (UDT), or sulfotransferases (SULT). Phase III: sequestration or compartmentation of the large, conjugated molecule into the plant cell wall or vacuoles out of harm’s way.^12^ Discoveries of plant enzymes involved in the degradation of xenobiotics, largely led by phytoremediation research, are today at the front line of research aiming to address the critical concern of nontarget site resistance (NTSR) in weeds.^13^

Environmental scientists and engineers had not much considered the power of catabolic enzymes in plants which have evolved through eons for detoxification, plant protection, secondary metabolite formation, and respiration.^14^ Biodegradation and catabolism were thought to be the domain of bacteria, fungi, and decomposers—not plants, the primary producers so ubiquitous on Earth. Some doubted that organic xenobiotic chemicals could pass through the membrane and Casparian strip of rooted plants to a sufficient extent to be uptaken and metabolized.

## Physical Chemistry and Plant Uptake

Burken and Schnoor began to study the physical chemistry necessary for the uptake of nonionic toxic organic chemicals.^15^ They reported that some chemicals were too hydrophobic and not bioavailable to plants for uptake, but there was a “sweet spot” in the range of log *K*_ow_ 1.5–4.^15^ Many chemicals could be uptaken by plants and without phytotoxicity.^16,17^ However, some chemicals were too toxic or insoluble (e.g., 2,4,6-trinitrotoluene, TNT) to allow a viable application of phytoremediation.^18^ In addition, of course, some groundwater contaminants were too deep in the subsurface (>15 ft bgs) for plant roots to access without pumping groundwater up to irrigate the root zone of plants. We concluded that for cleanup to be successful at a site, xenobiotic organic chemicals in the subsurface were required to be somewhat hydrophilic (bioavailable) and not too toxic and that it was necessary for roots to explore the entire contaminated zone for suitable mass transfer to occur.

## Microbial Processes in the Rhizosphere vs Plant Uptake

Somewhere along the way, we realized that phytoremediation for many neutral hydrophobic chemicals in soils occurred mainly in the rhizosphere by bacteria, not in the plant itself. Microbes found a suitable habitat in the root zone and were apparently aided by dissolved oxygen, exudates, and secondary metabolites leaked from plants.^19−21^ Exudates served as auxiliary substrates for cometabolism of aromatics like benzene/toluene/ethylbenzene/xylenes (BTEX), polynuclear aromatic hydrocarbons (PAHs), and long-chain alkanes in petroleum hydrocarbons.^21,22^ In some cases, exudates were inhibitory to metabolic degraders because they represented a readily bioavailable and degradable carbon source, causing diauxic or catabolic repression in the degradation of target compounds.^23,24^

Microzones and variable redox conditions allowed both aerobic and anoxic degradation pathways to exist in the same contaminated plant/soil systems for polychlorinated biphenyls (PCBs) phytoremediation.^25−27^ However, oxygen is critical for the aerobic degradation of total petroleum hydrocarbons (TPH) in the subsurface by phytoremediation.^19^ Often, a “smear zone” exists at former refineries and tank farm sites—an oily phase at a depth under low oxygen conditions through which roots cannot penetrate.^28^ Aerobic bacteria may express dioxygenase enzymes for the rapid oxidation of petroleum hydrocarbons in the root zone. We have seen hybrid poplar trees grow through pools of weathered surface oil but only if their root systems can track through zones of sufficient oxygen in soil gas to survive. Plant uptake and transformation of BTEX compounds may play a role in TPH phytoremediation, but it is mostly the bacteria in the rhizosphere that do the work. Dominant families include Actinobacteria, Proteobacteria, and Bacteriodetes. Long-term phytoremediation of petroleum hydrocarbons in soils at former tank farm sites has been successfully demonstrated.^29^

For several years, we tried to understand exactly what the plants were doing to biodegrade toxic organic chemicals versus the role that associated microbes played. We used various “controls” and attempted to poison the microbes to be able to observe solely plant biodegradation of chemicals in the absence of microbes. These attempts mostly failed because high concentrations of antibiotics or sterilizing agents were necessary to kill bacteria and fungi, but they also proved phytotoxic. Thus, we tried to raise sterile (axenic) plant tissues—callus, root, and shoot cultures. Apical meristems from plants were surface sterilized to serve as pluripotent stem cells (without bacteria and fungi). Despite our best efforts, we could not separate the role of microbes from the plants because it turns out that a whole world of microbes lives within the plant (as endophytes) and cannot be killed by surface sterilization! We found that ubiquitous bacteria and fungi lived in, on, and around plant leaves, shoots, and roots. Some were beneficial to the plant, protecting it from infection by microbes and protozoa, and some were opportunistic pathogens. Plant/microbe associations were often mutualistic, with microbes receiving substrates (food) from the plants and plants receiving minerals, nutrients, vitamins, or growth hormones from the microbes (auxins and cytokinins such as indole-3-acetic acid and *cis*-zeatin).

**Figure 2:**
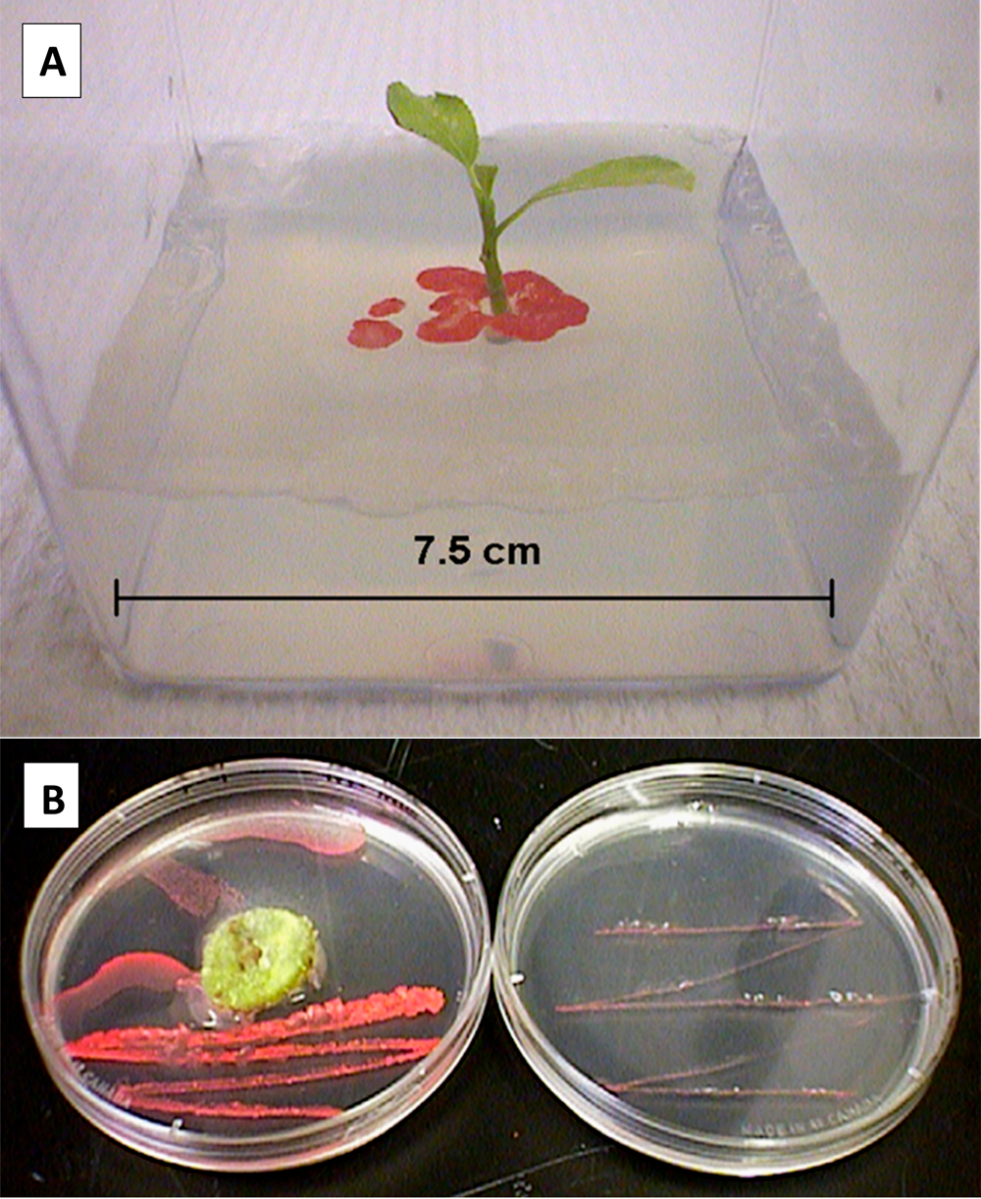
Inextricable mutualism between plants and attendant microbes. (A) Axenic poplar shoot culture growing in minimal agar with the leakage of the endophyte, *Methylobacterium populi*, emanating from inside the plant shoot onto the agar, revealing itself. (B) (Right) Agar plate shows the slow growth of *M. populi* on minimum agar. (Left) Plate shows how a small piece of living plant tissue (callus cell culture of *Populus deltoides* x *nigra*, DN34) provides carbon substrate (fructose) for vigorous growth of the endophyte bacteria, *M. populi*. In turn, the callus cells receive growth hormones (indole-3-acetic acid and *cis*-zeatin) from the bacteria.

Schnoor credits Benoit Van Aken, a postdoctoral and research scientist in our lab, with the “Eureka” moment in ca. 2004, “It’s the ecology, stupid!” That is when we discovered that our axenic shoot cultures (surface sterilized with alcohol) had multiple bacteria and fungi living inside (endophytes). In addition, some of the microbes were novel, never having been seen or documented before. Simply by serendipity, we stumbled upon *Methylobacterium populi* bacteria living inside hybrid poplar. We walked into the lab one morning to observe, to our surprise, that the shoot cultures displayed a pink-pigmented α-proteobacter shining brightly red on the surface of our agar (Figure 2). We never thought, as engineers, we would discover a new organism or publish it in a systematics journal, but we did!^30−32^ Our true discovery was that phytoengineers and scientists must learn to appreciate the entire plant/microbe/rhizosphere ecosystem to fully understand and apply phytoremediation.^33^ It is impossible to separate the role of plants versus microbes because they work together—it is all part of a mutualistic ecosystem, the microbiome. We stand transfixed by the remarkable coevolution that has accrued through time and its enzymatic transformative power to degrade xenobiotic contaminants.

Still, we lacked a fundamental understanding of plant transformations of toxic organic chemicals because catabolic processes had been ignored for so long. It turned out that hybrid poplar could, by itself, mineralize 1,3,5-trinitroperhydro-1,3,5-triazine (RDX) in plant tissues, although microbial processes by rhizosphere bacteria are likely faster.^34^ Plant uptake and biotransformation occur in parallel with rhizosphere biodegradation for many soluble compounds like ethers 1,4-dioxane, methyl-*tert*-butylether (MTBE), and explosives RDX and 1,3,5,7-tetranitro-1,3,5,7-tetraazacyclooctane (HMX).^35−39^ The powerful idea of coring or sampling plant tissues for phytoscreening of subsurface-contaminated zones (phytoforensics) was championed by Joel Burken and his students beginning in 2002.^40^ Sometimes, volatile organic chemicals like TCE could be transpired by poplar to the atmosphere, but many alkenes and VOCs are rapidly oxidized in the atmosphere by the cleansing power of hydroxyl radicals.^41^ Furthermore, some xenobiotic chemicals can be transformed within the translucent leaves with the aid of the sun, a.k.a. phytophotolysis.^42^

## Molecular Biology and Phyto

It became important to answer the following question: what genes encode for the plant enzymes necessary to biodegrade toxic organic chemicals? Regulatory authorities needed multiple lines of evidence and a documented explanation for how cleanup occurs in risk-based phytoremediation. *Arabidopsis* was the first plant whose genome was completely sequenced and appeared on a microarray chip around 2005.^43^ Affymetrix ATH1 GeneChip microarrays represented 22 810 genes of 7 *Arabidopsis thaliana* accessions.^44^ It was not long before the complete genome was available (but not well annotated) for *Populus trichocarpa*.^9^ Phytoresearchers began to use plant microarrays and real-time reverse-transcription PCR to determine which genes were up- or downregulated by common contaminants like TCE, BTEX, PCBs, petroleum hydrocarbons, pesticides, and explosive compounds. We then searched for the mRNA transcripts active in biodegradation (transcriptomics) and their translated enzymes (proteomics). Benoit Van Aken led the charge in our laboratory of this pursuit.^45^ Many phytoresearchers became involved with the “omics” revolution. We were fortunate to unravel some of the genes upregulated and involved in the transformation of 2,4-D and 2,4,5-T by *A. thaliana*(46,47) and RDX and TNT by poplar.^48,49^ Much of this molecular biology research with plants led us to explore improvements in the rhizosphere that could be achieved through bioaugmentation.^50^

## Bioaugmentation and Growth Factors

**Figure 3:**
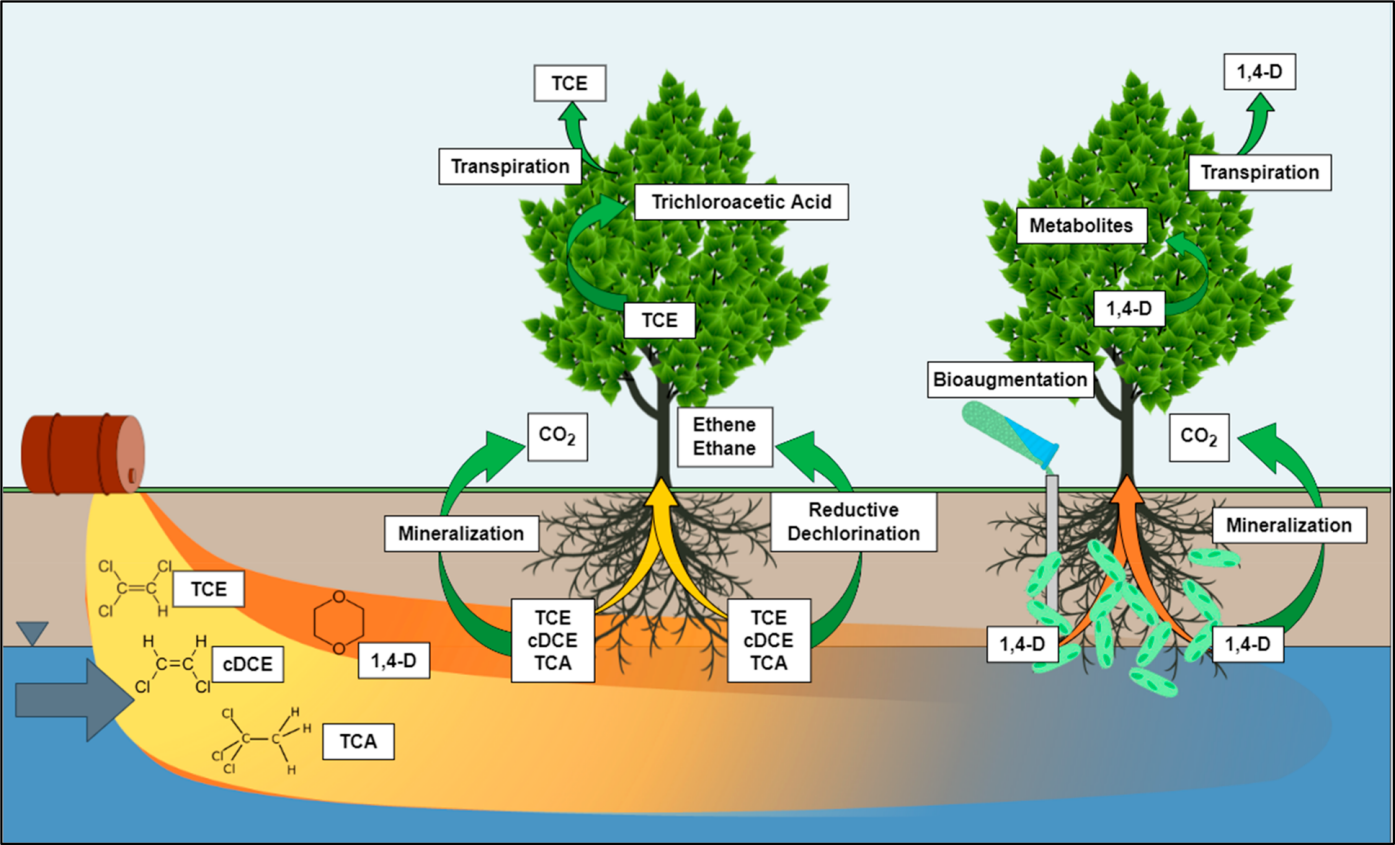
Concept of bioaugmented phytoremediation showing the synergy between the two technologies deployed together for the biodegradation of 1,4-dioxane and co-occurring chlorinated solvents, including trichloroethylene (TCE), *cis*-dichloroethylene (cDCE), 1,1,1-trichloroethane (TCA), and dichloroethane (DCA).

Recent studies have explored bioaugmented phytoremediation to enhance the treatment of xenobiotic compounds, including explosives,^32^ PCBs,^50^ chlorinated solvents,^51^ hydrocarbons,^52^ pesticides,^53^ and heavy metals.^54,55^ Building on our previous work,^35,36^ our recent efforts have focused on optimizing bioaugmentation of the poplar microbiome to treat 1,4-dioxane contamination. Because 1,4-dioxane was used as a stabilizer for chlorinated solvents, it is often found comingled with TCE, *cis*-DCE, TCA, and DCA.^56^ At some sites, the 1,4-dioxane plumes can reach for miles, possibly threatening the drinking water of nearby communities. Phytoremediation is well suited for these large and dilute plumes, where traditional remedial techniques are often prohibitively expensive. For example, poplars readily uptake and metabolize TCE into trichloroacetic acid (TCAA), dichloroacetic acid, and trichloroethanol (Figure 3).^57^ Anaerobic zones in the rhizosphere also allow for microbial reductive dechlorination of these solvents.^58^ 1,4-Dioxane is also readily uptaken by poplar, but due to its high miscibility in water (log *K*_ow_ = −0.27), the majority of dioxane (76.5 ± 3.9%) is transpired directly to the atmosphere.^35^ By bioaugmenting the poplar root zone with dioxane-degrading bacteria, we can increase dioxane metabolism in the rhizosphere and minimize the amount of dioxane transpired to the atmosphere (Figure 3).

During this work, we confirmed that through uptake and evapotranspiration, poplar trees alone can achieve low dioxane concentrations (∼1 μg/L) in the laboratory with simulated contaminated groundwater. We also demonstrated that bioaugmenting the rhizosphere with dioxane-metabolizing organisms, including *Pseudnocardia dioxanivorans* CB1190 and *Mycobacterium dioxanotrophicus* PH-06, speeds the treatment of dioxane.^59^ CB1190 can utilize root extract as an auxiliary carbon source, making it well equipped to colonize the poplar root zone.^36^ However, the root extract’s presence also caused catabolite repression in CB1190, slowing dioxane metabolism. In contrast, PH-06 cannot utilize root extract but was not sensitive to catabolite repression.

We also evaluated dozens of enrichment cultures and other dioxane-metabolizing organisms as bioaugmentation candidates for the poplar rhizosphere. We hypothesized that these strains required growth factors (i.e., amino acids or vitamins) not typically included in minimal microbial media. Through another stroke of serendipity, we discovered *Rhodococcus ruber* strain 219 rapidly grows on and degrades dioxane when supplemented with thiamine (vitamin B1).^60^ This strain had been previously reported only to grow very slowly on dioxane.^61^ In addition, when grown with thiamine, the strain had the fastest kinetics for dioxane metabolism reported to date. This discovery also underscored the complex syntrophic relationships between plants and microbes in the rhizosphere. Furthermore, many growth factors may be supplied by rhizospheric bacteria, fungi, or root exudates.^62−66^ Harnessing these syntrophic relationships is an important emerging area of research in bioaugmented phytoremediation. Engineering the microbiome of the plant rhizosphere is key to further progress and wider application of bioaugmented phytoremediation.

## Genetically Modified Plants, Bacteria, and Editing the Microbiome

Recent efforts have been made to bolster phytoremediation by optimizing plant cultivars through either conventional (traditional crossbreeding) or engineered (transgenics) techniques. The selective cultivation and screening of tree hybrids (e.g., *Populus* and *Salix* spp.) has shown promise in identifying genotypes and/or superior clones to maximize tree survival and remediation performance. This work can assist in selecting varieties best suited for the conditions and contaminants present at a site.^67,68^ Alternatively, transgenic plants have been produced by introducing exogenous genes to improve the uptake or metabolism of contaminants. For example, work by Doty et al. enhanced the treatment of TCE by inserting cytochrome P450 monooxygenases into poplar via a bacterial vector.^69^ Furthermore, Bruce et al. engineered *Arabidopsis*, tobacco, switchgrass, and wheatgrass to express a bacterial gene for phytoremediation of RDX.^70−74^ Transgenic plants have also been used to improve plant tolerance, uptake, and treatment of various metals, including lead, cadmium, mercury, and selenium.^75−78^ In addition, transgenic plants have been developed to excrete increased root exudates, in turn supporting increased biodegradation in the root zone.^79^

Another approach to enhance phytoremediation is through the use of genetically engineered bacteria. Successful bioaugmentation of the rhizosphere often hinges on strain survival postinoculation. This is often most successful if the bacteria are well adapted to the plant’s rhizosphere.^80^ However, isolating endophytic or rhizospheric bacteria capable of transforming contaminants has proven difficult. An alternative strategy is to engineer endophytic strains to express contaminant-degrading genes. This approach has been used to insert a toluene monooxygenase into plant-growth-promoting bacteria, *Burkholderia cepacia* VM1468.^81^ Subsequent inoculation into the poplar rhizosphere and exposure to toluene improved plant growth, decreased phytotoxicity, and enhanced toluene degradation in the root zone. However, upon closer investigation, the researchers could not detect or recover the initially inoculated strains. Instead, the inserted gene had horizontally transferred to various other endophytic strains. This remarkable unintended result highlights a potential added benefit to bioaugmented phytoremediation. One of the strains which received the horizontally transferred toluene gene has also been used to enhance degradation of TCE in the poplar rhizosphere.^82,83^ Genetically modified endophytes have also been used to improve tolerance and uptake of nickel.^84^

An alternative strategy to improve inoculated strain survival is to engineer the plant microbiome before inoculation. Because the plant microbiome can support a highly diverse microbial community, this can increase competition for inoculated strains.^85^ For example, desirable bioaugmented strains may not effectively colonize the rhizosphere due to competition for required resources (e.g., carbon source, oxygen, growth factors). To better understand plant–microbe interactions, researchers have utilized metagenomic analyses to ensure the colonization and survival of inoculated strains.^86^ Other techniques suggest altering selective pressures to improve strain survival.^85^ Recent progress has also been made in “niche clearing” using bacteriophages.^87^ Employing this technique, researchers can perform precise microbiome editing, allowing for the suppression of specific microbial activities, which may open niches for inoculated strains. Furthermore, this technique may improve various plant traits, such as increased drought and disease resistance. Other emerging technologies, such as specific gene editing using CRISPR, will streamline the engineering of plants, endophytes, and the plant microbiome, opening new frontiers for phytoremediation research.

The future outlook for phytoremediation and its various off-shoots is indeed bright. Applications to emerging contaminants seem likely, such as per- and polyfluoroalkyl substances (PFAS), brominated and organophosphate flame retardants, synthetic musks and other personal care product ingredients, industrial chemical additives, stabilizers, adjuvants, and hormonally active compounds. Natural treatment systems for water and wastewater (green infrastructure) with low carbon footprints are expanding rapidly and have gained the confidence of consulting engineers. Low-impact development (LID) to slow down stormwater, which improves infiltration and groundwater recharge, often employs plant-based strategies. Constructed wetlands, floating mats, rain gardens, bioswales, green roofs, and riparian zone buffer strips are gaining acceptance for improvement of water quality and LID. Increasingly, architects and landscapers utilize green walls on buildings and plants for indoor air filtration and purification systems. Phytoremediation can clean water, air, and soil in a cost-effective, natural green system.

Probably the greatest environmental challenge facing humanity is how to stabilize the chemistry of our atmosphere and control climate change. Massive plantations of native trees and grasses on previously degraded marginal lands are a potent and cost-effective climate solution. It is already underway as a Great Green Wall by China in the Gobi Desert and as an African Union Project in the Sahel in Sub-Saharan Africa. Replanting temperate and boreal forests worldwide could remove CO_2_ from the atmosphere (negative emissions) and restore organic carbon to soils. When forests reach climax, they could be harvested for products like biochar to sequester carbon long term as a soil conditioner and applied on land to facilitate replanting and regrowing the forests as a long-lasting solution.
